# Ellagic acid alleviates NLRP6/caspase-1/GSDMD-mediated inflammation and pyroptosis in rats post cerebral ischemia/reperfusion injury

**DOI:** 10.22038/ijbms.2024.78864.17057

**Published:** 2025

**Authors:** Ling Hu, Xiaoqiong Wei, Guofu Shen, Xiaohuan Huang

**Affiliations:** 1 Department of Pathology, Chongqing Three Gorges Medical College, Wanzhou, China; 2 Chongqing Three Gorges Medical College, Wanzhou, China

**Keywords:** Cerebral ischemia/reperfusion injury, Ellagic acid, Inflammation, NLRP6 inflammasome Pyroptosis

## Abstract

**Objective(s):**

Ellagic acid (EA) is a natural polyphenol with anti-cancer, anti-oxidant, anti-inflammatory, antibacterial, and other effects. However, the role of EA in cerebral ischemia/reperfusion injury (CIRI) remains unclear. This study aims to investigate the neuroprotective effects of EA in CIRI.

**Materials and Methods:**

Forty male Wistar rats (260-300 g) were randomly divided into four groups with 10 rats per group: 1) Sham+Veh: Rats underwent I/R surgery, except that they were not inserted with thread plugs, and received solute treatment at the same time. 2) MCAO/R+Veh. 3) MCAO/R+EA: Rats were administered 200 mg/kg EA before undergoing MCAO. 4) MCAO/R+Nim: Rats were administered Nim before undergoing MCAO.

**Results:**

Cerebral MCAO/R damaged brain tissue, elevated neurological deficit score (*P<*0.01), cerebral infarction volume (*P<*0.01), inflammatory cell infiltration (*P<*0.01), NLRP6, ASC, caspase-1 and GSDMD mRNA level (*P<*0.01 and *P<*0.001), NLRP6, caspase-1, GSDMD-N and IL-1β protein level (*P<*0.01 and *P<*0.001), and inflammatory cytokines in brain tissue (*P<*0.01). Prophylactic administration of EA also significantly improved brain tissue damage, reduced neurological deficit score (*P<*0.01), cerebral infarction volume (*P<*0.01), inflammatory cell number (*P<*0.05), NLRP6, caspase-1, GSDMD-N mRNA and protein level (*P<*0.05 and *P<*0.01), ASC mRNA level and IL-1β protein level (*P<*0.01), and IL-1β and IL-18 level in brain tissue (*P<*0.01) compared to positive control.

**Conclusion:**

EA may serve as a potential drug for the treatment of brain I/R, which may exert an anti-inflammatory effect by inhibiting the activation of the inflammasome.

## Introduction

Ischemic stroke is a serious global problem with a high incidence rate, mortality, and recurrence rate (1, 2). Cerebral ischemia-reperfusion injury (CIRI) is a key pathological process that exacerbates neurological damage in ischemic stroke patients (3), including calcium overload (4), free radical damage (5), lipid peroxidation (6), inflammatory response (7), and excitatory amino acid toxicity (8). Studies have shown that inflammatory responses promote the development of CIRI, among which neuronal apoptosis has been proven to be closely related to inflammatory response (9). As part of the pattern recognition receptor family, the NOD-like receptor (NLR) family can recognize PAMPs in the cytoplasm (10) and DAMPs in the cytoplasm after tissue damage or cell death. As a member of the NLR family, nucleotide-binding oligomerization domain like receptor protein 6 (NLRP6) participates in the formation of inflammatory complexes during CIRI, consisting of NLRP6, apoptosis-associated speckled protein (ASC), and aspartate cysteine specific protease-1 (11). The activation of the NLRP6 inflammatory complex can promote the release of pro-inflammatory factors, thereby exerting pro-inflammatory effects (12).

Therefore, the use of natural substances or herbs with anti-inflammatory properties to improve lesions in neurological diseases such as Alzheimer’s disease (13), Parkinson’s disease (14), and ischemic stroke (15) has become a current research focus. They may protect the brain through a mechanism that reduces nerve damage after a stroke (16).

Ellagic acid is a derivative of gallic acid and a natural polyphenol widely present in natural plants, especially in plants such as pomegranates, grapes, strawberries, Chinese tallow, fupan, licorice, and digitalis. At present, Ellagic acid plays an important role in the prevention and treatment of diseases through anti-inflammatory (17), antibacterial (18), antiviral (19), anti-tumor (20), hypoglycemic (21), lipid-lowering (22), anti-atherosclerosis (23), osteoporosis prevention (24), and immune regulation (25) properties. However, the mechanism of ellagic acid affecting cerebral I/R injury is not fully understood and remains to be confirmed. 

Regarding the neuroprotective effects of natural drugs on ischemic stroke, we focused on the preventive, protective effect of EA on inflammation and pyrosis caused by cerebral I/R injury. After a rat I/R model, neurological dysfunction and inflammatory cell infiltration occur. Therefore, this study investigated brain tissue injury, inflammasome activation, and pyroptosis of nerve cells in rats with cerebral I/R.

## Materials and Methods

***Animals and experimental protocols***

Eighty-eight male SD rats, 8-10 weeks old, 260-300 g, were provided by Hunan Slake Jingda Experimental Animal Co., Ltd. The rats were kept at a temperature of 24±2 ^°^C and a humidity of approximately 60%. They were adaptively raised for one week and allowed to eat and drink water freely. This process was conducted in accordance with the Chongqing Three Gorges Medical College ethics committee approval code of ethics for all animals. (SXYZ-A-202401-0001).

**Figure 1:**
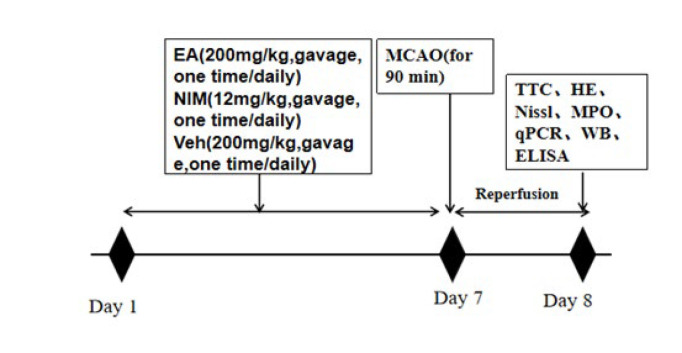
Illustrative diagram of experimental protocols for different drug treatment groups

MCAO: middle cerebral artery occlusion; EA: Ellagic acid; NIM: Nimodipine; Veh: Vehicle; TTC: 2,3,5-Triphenyltertrazolium chloride; HE: Hematoxylin-eosin; MPO: Myeloperoxidase; qPCR: Quantitative Polymerase Chain Reaction; ELISA: Enzyme linked immunosorbent assay

Animals were divided randomly into four groups: ① Sham+Veh group. The only difference was that the left common carotid artery was not blocked, the other surgical methods were the same as those in the model group, and 1% Sodium carboxymethyl cellulose was used as the solvent control of EA once a day for seven days. ②MCAO/R+Veh group: MCAO/R rats received sodium carboxymethyl cellulose (1%) once a day for seven days. ③MCAO/R+EA groups: MCAO/R rats received 200 mg/kg EA once daily for one week. ④Positive control: MCAO/R rats received 12mg/kg Nim once daily for one week. All the drugs were prepared with 1% sodium carboxymethyl cellulose and orally administrated. The timeline and experimental design are shown in Figure 1.

***MCAO model***

MCAO surgery was performed in rats under anesthesia (3.5% chloral hydrate, 1 ml/100 g) as previously described (26). Take the supine position and cut at the midline of the neck. A “v” shaped incision was made in the left common carotid artery. Nylon monofilament was inserted into the blood vessel, through the left external carotid artery, and finally into the middle cerebral artery (black mark over the bifurcation). After 90 min, the nylon monofilament was removed, and the rats were treated after 24 hr of reperfusion. The sham group underwent the same operation as the MCAO group, except that no nylon monofilament was inserted.

***Cerebral infarction volume assessment***

In order to determine the volume of the cerebral infarction, the whole brain of the rat was removed and frozen at -20 ^°^C for half an hour, and the brain was sliced every 2 mm (a total of 5 pieces) as previously reported. The slices were then rapidly placed in 2% 2,3, 5-triphenyltetrazepine ammonium chloride (TTC)(37 ^°^C; 15 min); this step requires light protection, and the TTC needs to be heated in a 37 ^°^C water bath in advance. Finally, the slices were transferred to 4% paraformaldehyde and fixed for 48 hr. The staining results were captured using a digital camera, and infarct volume analysis was performed using the ImageJ software. The infarct volume is calculated as follows:{[total lesion volume-(ipilateral hemisphere volume - contralateral hemisphere volume)]/contralateral hemisphere volume}× 100% (27).

***Evaluation of neurological score***

According to the previous literature (28), 24 hr after modeling, the neurological damage of rats was scored as follows :0, normal; 1, The opposite forelimb cannot be fully extended; 2, Turn to the opposite side (turn round) during movement; 3, Pour to the opposite side; 4, cannot move autonomously; 5, Death. Rats with scores of 0 and 5 should be excluded.

***Hematoxylin-eosin (HE) and Nissl staining***

According to previous literature (29, 30), after MCAO, the brain tissue was rapidly collected and fixed in 4% paraformaldehyde for approximately 24 hr. Next, the brain tissue, about 2 mm thick, was taken along the coronal axis. It was dehydrated, made transparent, waxed, embedded, sliced, retrieved, baked, stained, and sealed with HE and Nizl, then observed under a microscope.

***Myeloperoxidase (MPO) staining***

Rat brain tissue was placed in 4% paraformaldehyde internal fixation for 24 hr and dehydrated with sucrose gradient. Then, the brain tissue was cut into 4~5 μm slices using a frozen microtome and stained with myeloperoxidase antibody. The staining results were observed under a fluorescence microscope (31).

***Quantitative RT-PCR***

Total RNA was extracted from fresh rat cortical tissues of each group (32), and the RNA concentration in each group was determined using a NanoDrop 2000 ultraviolet spectrophotometer. According to the determined RNA concentration, the reverse transcription reaction system was carried out using All-In-One 5X RT MasterMix, and the reaction conditions were 37 ^°^C for 15 min, 60 ^°^C for 10 min, 95 ^°^C for 3 min, and corresponding cDNA was obtained. BlasTaqTM 2X qPCR MasterMix was used for PCR amplification at 95 ^°^C for 3 min. 95 ^°^C for 15 sec, 60 ^°^C for 1 min, 40 cycles. Using β-actin as an internal reference, the relative expression of β-actin was analyzed by 2^−ΔΔCt^. The sequence of primers is shown in Table 1.

***Western blot***

We took cerebral cortex tissues from rats, weighed them, and added freshly prepared RIPA lysate containing PMSF. Then, we ground the mixture on ice using a hand-held grinder, let it sit on ice for 30 min at 4 ^°^C, centrifuged at 12,000 rpm for 15 min, and removed the supernatant. For each concentration measurement, we used the BCA method and heated it to 99.5 ^°^C for 5 min in a metal bath for denaturation. The electrophoresis process began immediately after loading the sample. The gel was then cut, and the protein was transferred to a PVDF membrane using a constant current. The membrane transfer time was determined based on the molecular weight. Non-specific antigens were blocked, and then the membrane was kept at 4 ^°^C overnight. During this time, it was incubated with rabbit anti-NLRP6, caspase-1, GSDMD, GSDMD-N, and IL-1β, all at a concentration of 1:1000. After that, corresponding secondary antibodies were applied at room temperature for 1–2 hr. Then, ECL chemiluminescence was performed using β-actin as the internal reference. ImageJ software was used to conduct a semi-quantitative analysis of each group of bands, and the gray value of the target protein was used as the numerator, and the gray value of the internal reference band was used as the denominator to calculate the relative expression of the target protein.

***Measurement of cytokine levels in brain tissue***

The supernatant was obtained after extracting the protein from the brain tissue of rats in each group. This was achieved by taking the sample diluent of IL-1β and IL-18 ELISA kits, diluting the supernatant at a ratio of 1:10, and then setting up blank wells (without adding samples and enzyme-labeled reagents), standard products, and sample wells to be measured. We added the sample diluent, each concentration standard, and 40 ul and 10 ul of the sample diluent to be tested. In turn, we incubated the mixture at 37 ^°^C for 30 min. After washing again, we added 50 ul of color developers A and B successively, mixed gently, and allowed the color to develop at 37 ^°^C for 10 min away from light. After that, we added 50 ul of termination liquid to each well to terminate the reaction. The OD value of each hole is measured at 450 nm with blank hole zeroing.

***Statistical analysis***

SPSS 25.0 software was used to process the data, and the results were expressed as the mean±SEM. Normal distribution and homogeneity tests were used for statistical significance analysis, and one-way analysis of variance was used for multiple comparisons. *P*<0.05 was considered to indicate statistical difference.

## Results

In this study, we selected EA (200 mg/kg) as the most effective dose with reference to previous literature reports (33) and preliminary experiments and Nim (12 mg/kg) as the positive control with reference to previous literature (34).

***Neurological deficit scores***

**Figure 2:**
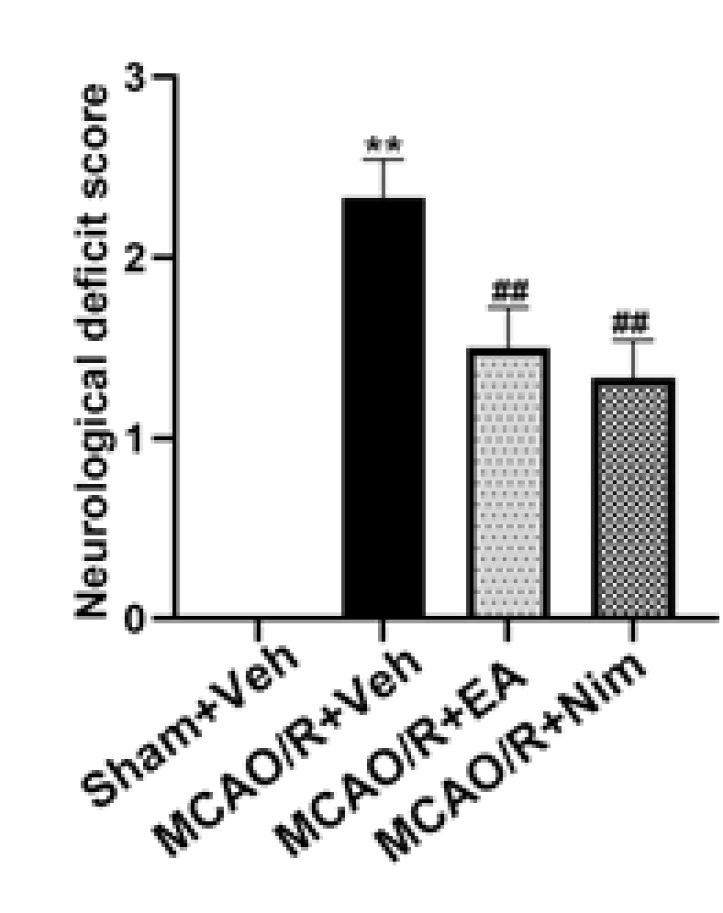
Neurological function score of rats with cerebral ischemia for 2h/reperfusion for 24h afterward

The score results obtained from the neural function score are shown in Figure 2. The MCAO/R+Veh group showed a significant increase in the neurological deficit score compared to the Sham+Veh group (*P<*0.01). Both the EA and positive control group could significantly improve clinical symptoms, and compared with the MCAO/R+Veh group, the neurological deficit scores of the EA and positive control group were significantly reduced (*P*<0.01). The results suggest that EA could alleviate neurological impairment.

***Cerebral infarction volume***

**Figure 3:**
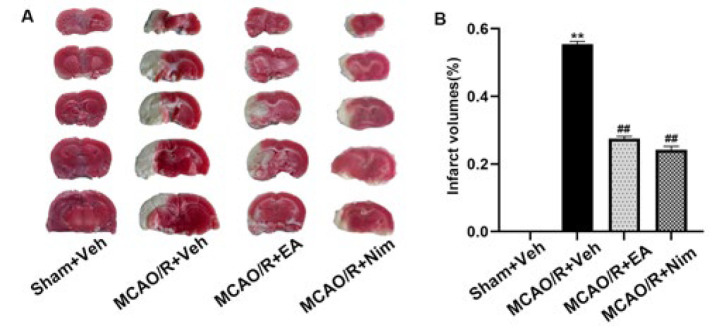
Evaluation of cerebral infarction volume (A-B) with 2,3, 5-triphenyltetrazepine ammonium chloride (TTC)

Figure 3A shows images of cerebral infarction in different groups. Figure 3B shows the statistics of infarct volume in different groups. The MCAO/R+Veh group was significantly larger than the Sham+Veh group (*P*<0.01). Compared with the MCAO/R+Veh group, the infarct volume was significantly reduced in the EA group and the positive control group (*P*<0.01). The result suggested EA alleviated cerebral impairment.

***Pathological change***

**Figure 4:**
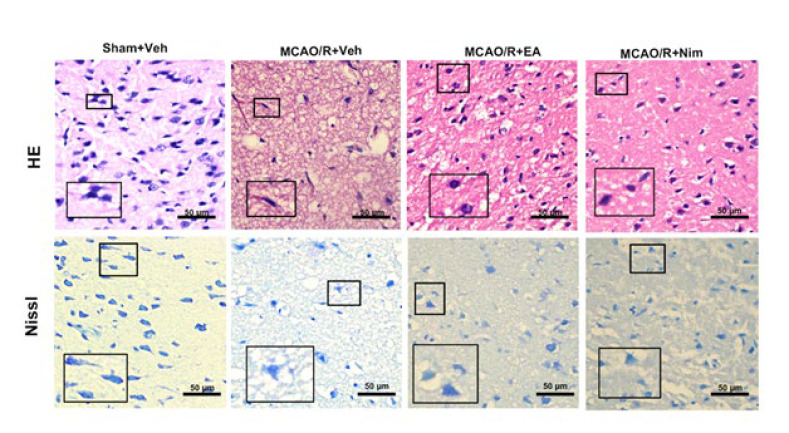
Evaluation of pathological alterations and neuronal injury in the brain tissue of rats with HE and Nissl staining

HE: Hematoxylin-eosin; MCAO/R: middle cerebral artery occlusion/reperfusion; Veh: vehicle; EA: Ellagic acid; NIM: Nimodipine

Figure 4A shows the morphological changes of the rat cortex in all tested groups. Compared to the Sham+Veh group, the brain tissue of the MCAO/R+Veh group showed significant edema, decreased numbers of neurons, irregular morphology, increased perinuclear vacuoles, cell swelling, and a large number of inflammatory cells in the stroma. Compared with the MCAO/R+Veh group, the above lesions in the cortical brain tissue of rats in the EA group and positive control group were alleviated to varying degrees, showing a tighter brain tissue structure, a decrease in the number of condensed nuclei, and a reduction in inflammatory cells. Figure 4B shows the pathological changes of neurons. Compared with the Sham+Veh group, the atrophy of Nissl bodies in the cytoplasm of neurons in the MCAO/R+Veh group was significant; compared with the MCAO/R+Veh group, the atrophy number of Nissl bodies in the EA group and the positive control group was significantly alleviated. These findings suggest that EA could alleviate brain injuries.

***Infiltration of inflammatory cells***

**Figure 5:**
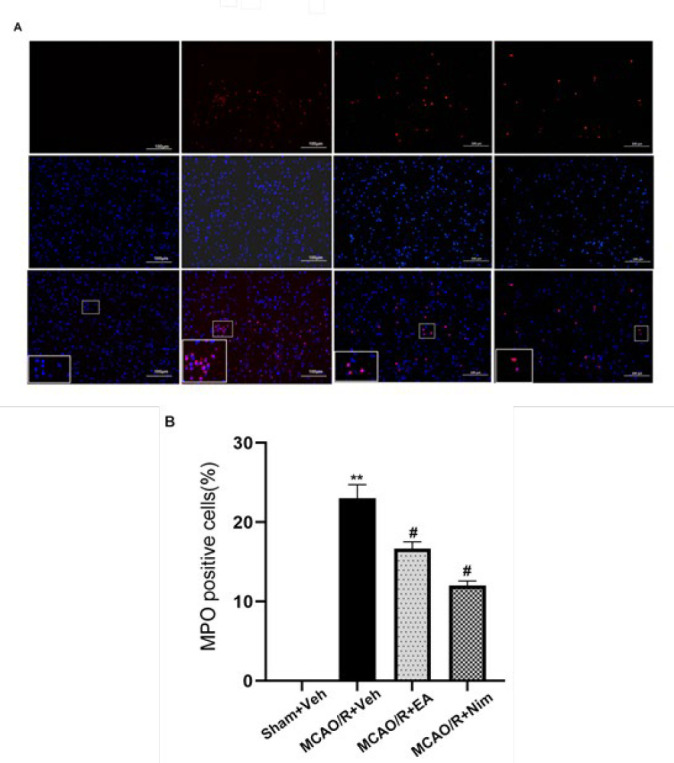
Evaluation of inflammatory cell infiltration of the left cerebral cortex ischemic penumbra area with MPO staining (A-B)

Figure 5A-B illustrates the infiltration of inflammatory cells in the cerebral cortex. Compared with the Sham+Veh group, the MCAO/R+Veh group was significantly increased (*P*<0.01)(5A-B). Compared with the MCAO/R+Veh group, the number of inflammatory cells was significantly reduced in the EA group and the positive control group (*P*<0.05)(5A-B). The results suggest that EA could alleviate inflammatory response following cerebral I/R injury.

***Cerebral cortex NLRP6, ASC, caspase-1 and GSDMD mRNA level***

**Figure 6:**
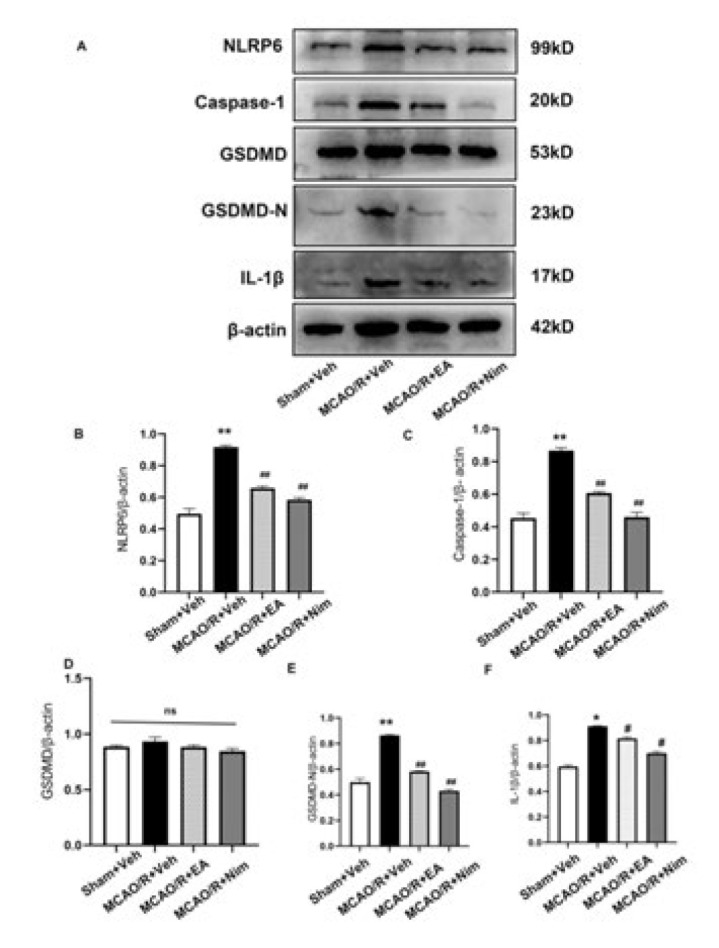
Evaluation of expression of mRNA of NLRP6 (A), ASC (B), caspase-1 (C), and GSDMD (D) with RT-PCR

Figure 6 (A-D) shows the mRNA expression of NLRP6, ASC, caspase-1 and GSDMD in the cerebral cortex. Compared with Sham+Veh group, the mRNA levels of NLRP6, ASC, caspase-1, and GSDMD in cortex were significantly increased in MCAO/R+Veh group (*P*<0.01 and *P*<0.001); Compared with MCAO/R+Veh group, the expression of NLRP6, ASC, caspase-1 and GSDMD mRNA in EA group and positive control group were significantly decreased (*P*<0.01 and P<0.001). In conclusion, EA could influence the mRNA level of NLRP6 inflammasome during cerebral I/R injury.

***Cerebral cortex NLRP6, caspase-1, GSDMD, GSDMD-N, and IL-1β protein level***

**Figure 7:**
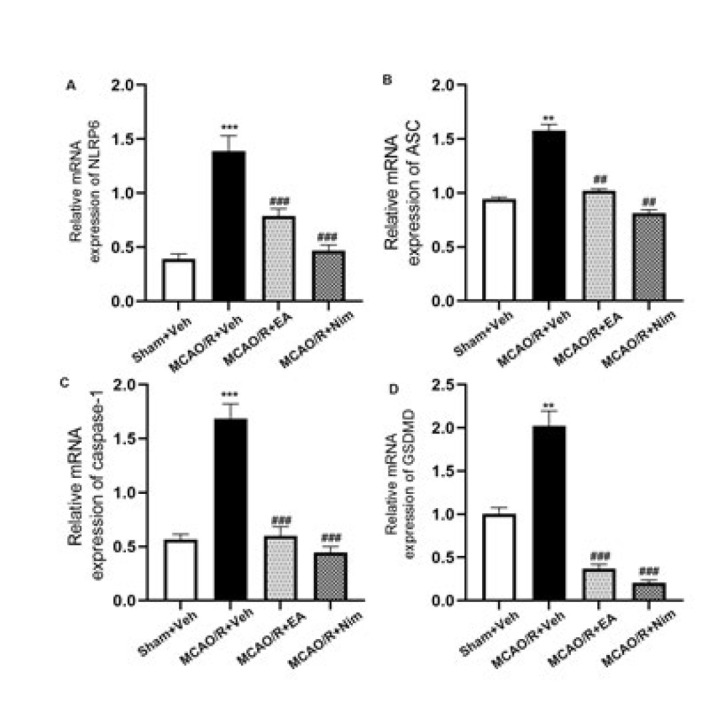
Evaluation of the expression of NLRP6, caspase-1, GSDMD, and IL-1β proteins with Western blot (A-F)

Figure 7 (A-F) shows the protein level of the cerebral cortex (A) and semi-quantitative statistics (B-D, F) in all experimental groups. The protein levels of NLRP6, caspase-1, GSDMD-N, and IL-1β were significantly elevated in MCAO/R+Veh group compared to the Sham+Veh group (*P*<0.05 and *P*<0.01). Compared with the MCAO/R+Veh group, the protein levels of NLRP6, caspase-1, GSDMD-N, and IL-1β in the cortex of the EA group and positive control group significantly decreased (*P*<0.05 and *P*<0.01). There was no statistical difference in the expression of GSDMD among the groups (E). In conclusion, EA could affect the protein level of inflammation and pyroptosis during cerebral I/R injury.

***Cerebral cortex IL-1β and IL-18 level***

**Figure 8:**
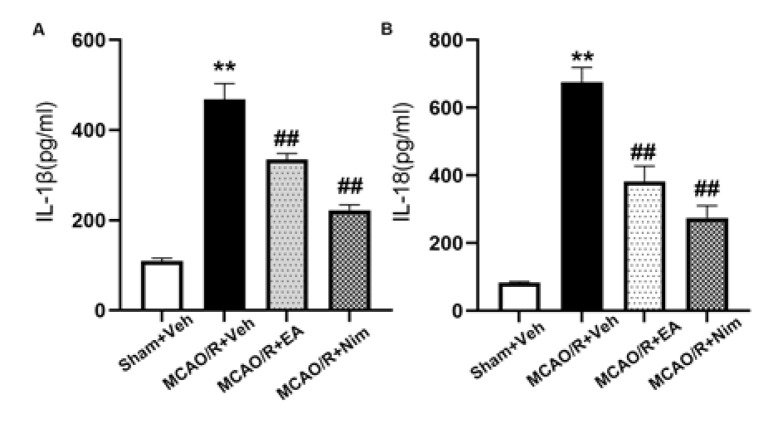
The expression levels of IL-1β (A) and IL-18 (B) in the left cerebral cortex ischemic penumbra area following ischemia/reperfusion with ELISA

Figures 8A and 8B show the expression levels of IL-1β and IL-18 in the cerebral cortical tissue. The expressions of IL-1β and IL-18 in MCAO/R+Veh group were significantly higher than those in Sham+Veh group (*P*<0.01). The EA group and positive control group were significantly decreased compared to the MCAO/R+Veh group (*P*<0.01). The results showed that EA could affect the release of inflammatory factors.

**Table 1:** Sequences of the primers for NLRP6, ASC, and Caspase-1

Name	Primer sequence	Product (bp)
NLRP6	Forward: 5′-CGCATCGTCTACTGTTCATCCTG-3′	112
	Reverse: 5′-AGCACTCTCAAGCCACTCGTAG-3′	
ASC	Forward: 5′-ATGGAAGAGTCTGGAGCTGTGG-3′	99
	Reverse: 5′-AATGAGTGCTTGCCTGTGTTGG-3′	
Caspase-1	Forward: 5′-TTGCCCTTTAGAAATAGCCCAGAAG-3′	150
	Reverse: 5′-TCAACATCAGCTCCGACTCTCC-3′	
β-actin	Forward: 5′-TGTCACCAACTGGGACGATA-3′	150
	Reverse: 5′-GGGGTGTTGAAGGTCTCAAA-3′	

**P<0.01 vs sham+Veh group. ^##^*P<*0.01 vs MCAO/R +Veh group

MCAO/R: middle cerebral artery occlusion/reperfusion, Veh: vehicle, EA: Ellagic acid, NIM: Nimodipine

ASC: Apoptosis-associated speck-like protein; NLRP6: Nod-like receptor protein six; Caspase- 1: Cysteinyl aspartate specific Proteinase- 1

Data were represented as Mean±SEM (n=6). **P<0.01 vs sham+Veh group. ^##^*P<*0.01 vs MCAO/R +Veh group

MCAO/R: middle cerebral artery occlusion/reperfusion, Veh: vehicle, EA: Ellagic acid, NIM: Nimodipine

Data were represented as Mean±SEM (n=6). **P<0.01 vs sham+Veh group. #*P<*0.05 vs MCAO/R +Veh group

MPO: Myeloperoxidase, MCAO/R: middle cerebral artery occlusion/ reperfusion, Veh: vehicle, EA: Ellagic acid, NIM: Nimodipine

Data were represented as Mean±SEM (n=6). **P<0.01, ***P<0.001vs sham+Veh group. ^##^*P<*0.01, ^###^*P<*0.001vs MCAO/R +Veh group

MCAO/R: middle cerebral artery occlusion/reperfusion; Veh: vehicle; EA: Ellagic acid; NIM: Nimodipine

Data were represented as Mean±SEM (n=6). **P<0.01, *P<0.05 vs sham+Veh group. ^##^*P<*0.01, #*P<*0.05 vs MCAO/R +Veh group

MCAO/R: middle cerebral artery occlusion/reperfusion; Veh: vehicle; EA: Ellagic acid; NIM: Nimodipine; ns: not statistically

Data were represented as Mean±SEM (n=6). **P<0.01 vs sham+Veh group. ^##^*P<*0.01, vs MCAO/R +Veh group

IL-1β; Interleukin-1 beta, IL-18; Interleukin-18, MCAO/R; middle cerebral artery occlusion/reperfusion, Veh: vehicle, EA: Ellagic acid, NIM: Nimodipine. ns; not statistically

## Discussion

Studies have shown that cerebral ischemia/reperfusion injury caused by middle cerebral artery embolization promotes NLRP6 inflammasome activation (35), leading to the occurrence of neuronal pyroptosis (36), accompanied by the release of a large number of pro-inflammatory cytokines. However, the use of EA (200 mg/kg) one week before cerebral ischemia/reperfusion injury can reduce inflammation related indicators.

Ischemic stroke (37, 38) is a common cause of disability and death, seriously endangering human life and health. Early reperfusion is crucial for blood flow recovery in ischemic brain tissue. However, a series of complex pathophysiological changes will occur in brain tissue after reperfusion, further aggravating cell injury and death (39). The inflammatory cascade (40) plays a key role. It has been found that the inflammatory response (41) is initiated by recognition of acute injury by pattern recognition receptors, both intracellular and extracellular. These receptors appear within hours after injury and can recognize pathogen-associated molecular patterns (PAMPs) or damage-associated molecular patterns (DAMPs), activate downstream inflammatory signaling pathways, and produce tumor necrosis factor-α (TNF-α), Interleukin 6 (IL-6), interleukin 1β (IL-1β) (42), and other pro-inflammatory factors and chemokines, which further aggravate the inflammatory response and eventually lead to nerve injury. Our results show that EA can reduce the production of inflammatory factors in cerebral ischemia/reperfusion injury.

As one of the pattern recognition receptors, NOD-like receptors (NLRs) can sense extracellular and intracellular stressors under inflammatory stimulation and can form a multi-protein complex with apoptosis-associated speckle protein (ASC) and caspase-1 or caspase-11 during DAMPs-mediated activation. This complex is called the inflammasome (43). Through inflammasome assembly, Pro-Caspase-1 is activated into cleaved-caspase-1, and Pro-IL-1β or Pro-IL-18 is cleaved into mature IL-1β or IL-18 (44), respectively, thus inducing an inflammatory immune response in response to cell damage. Simultaneously, activated caspase-1 triggers the processing of Gasdermin D (GSDMD) (45), which results in the formation of pores on the affected cell membrane. This leads to the release of GSDMD and IL-1β or IL-18, collectively promoting pyroptosis. Pyroptosis, in turn, initiates inflammatory cascades and ultimately causes nerve damage. The current research results show that EA can reduce the neurological function score and infarct volume, indicating that EA can alleviate neurological injury. The inhibition of inflammatory cytokines and pyroptosis may mediate the neuroprotective effect of EA.

As a member of the NOD-like receptor family, NLRP6 consists of three parts: N-terminal PYD domain, central NACHT domain (shared by all NLRS), and C-terminal leucine-rich repeat (LRR). In some cases, NLRP6 recruits apoptotic associated protein with serine/threonine kinase 1 (ASC) and caspase-1 or caspase-11 to form the NLRP6 inflammasome after activation, which mediates the maturation and secretion of pro-inflammatory cytokines IL-18 and IL-1β (46). The study’s results suggest that EA may alleviate inflammatory response and pyroptosis by inhibiting the activation of NLRP6 inflammasome.

EA has the effects of improving inflammation, inhibiting cell apoptosis, and clearing free radicals. It has been shown that ellagic acid can significantly down-regulate the levels of liver inflammatory factors NF-κB, TNF-α, IL-1β, and IL-6 in mice with fatty liver disease induced by AKT gene transfection (47). It may exert its anti-inflammatory effect through the LPS-TLR4-NF-κB pathway. In the inflammatory model of human fibroblast synoviocytes MH7A induced by TNF-α, intervention with ellagic acid was found to down-regulate the MTA1/HDAC1 complex in MH7A cells, promote apoptosis in MH7A cells, and inhibit inflammation (48). *In vivo* experiments have shown that ellagic acid can significantly improve the blood-brain barrier, brain tissue damage, and motor and exploratory behavior in rats with cerebral ischemia/reperfusion, effectively inhibiting inflammatory response (49). 

## Conclusion

Inflammatory cascades exacerbate cerebral ischemia/reperfusion injury. Activation of inflammasomes, release of inflammatory factors, and pyroptosis result in significant neurological damage. EA is a promising therapeutic agent with minimal side effects for cerebral I/R injury. However, the exact mechanism of EA treatment for cerebral ischemia-reperfusion injury requires further investigation.

## Declaration

We have not used any AI tools or technologies to prepare this manuscript.
